# Aerodynamic roughness variation with vegetation: analysis in a suburban neighbourhood and a city park

**DOI:** 10.1007/s11252-017-0710-1

**Published:** 2017-11-27

**Authors:** Christoph W. Kent, Keunmin Lee, Helen C. Ward, Je-Woo Hong, Jinkyu Hong, David Gatey, Sue Grimmond

**Affiliations:** 1 Department of Meteorology, University of Reading, Reading, RG6 6BB UK; 2 Ecosystem-Atmosphere Process Laboratory, Department of Atmospheric Sciences, Yonsei University, Seoul, 03722 Republic of Korea; 3 Risk Management Solutions, London, EC3R 8NB UK

**Keywords:** Anemometric methods, Aerodynamic roughness length, Morphometric methods, Source area, Vegetation, Wind, Zero-plane displacement

## Abstract

Local aerodynamic roughness parameters (zero-plane displacement, *z*_*d*_, and aerodynamic roughness length, *z*_*0*_) are determined for an urban park and a suburban neighbourhood with a new morphometric parameterisation that includes vegetation. Inter-seasonal analysis at the urban park demonstrates *z*_*d*_ determined with two anemometric methods is responsive to vegetation state and is 1–4 m greater during leaf-on periods. The seasonal change and directional variability in the magnitude of *z*_*d*_ is reproduced by the morphometric methods, which also indicate *z*_*0*_ can be more than halved during leaf-on periods. In the suburban neighbourhood during leaf-on, the anemometric and morphometric methods have similar directional variability for both *z*_*d*_ and *z*_*0*_. Wind speeds at approximately 3 times the average roughness-element height are estimated most accurately when using a morphometric method which considers roughness-element height variability. Inclusion of vegetation in the morphometric parameterisation improves wind-speed estimation in all cases. Results indicate that the influence of both vegetation and roughness-element height variability are important for accurate determination of local aerodynamic parameters and the associated wind-speed estimates.

## Introduction

The (dis)services of urban vegetation are both context and scale specific, therefore cannot be generalised (Salmond et al. 2016). However, as the socio-environmental and economic benefits of urban ‘green spaces’ are realised, they are increasingly becoming part of planning agendas to mitigate climate change, improve urban sustainability and improve human well-being (e.g. Gill et al. 2007; Landry and Chakraborty 2009; Roy et al. 2012; Andersson-Sköld et al. 2015; Kremer et al. 2015; Salmond et al. 2016; Ward and Grimmond 2017). Green spaces therefore will continue to be (a greater) part of the urban fabric. Despite this, when modelling the urban environment vegetation is often neglected to simplify the problem (e.g. references within Grimmond et al. 2010, 2011). It is imperative that the understanding of the physical implications of urban vegetation is improved across micro-, local-, and regional scales. This extends beyond urban parks and vegetation in street canyons – as the edges of cities are approached vegetation may become the most prominent roughness elements (e.g. Giometto et al. 2017, Kent et al. 2017a).

The presence of urban vegetation has implications for the storage and fluxes of scalar properties (e.g. heat, moisture and pollutants). For example, vegetation can reduce the mean and extreme ambient and indoor temperatures (Smith et al. 2011, Schubert et al. 2012, Mavrogianni et al. 2014, Heaviside et al. 2015), whilst also reducing night-time longwave cooling (Coutts et al. 2016). Its presence tends to increase humidity (through increasing evapotranspiration) and is also responsible for precipitation interception, a reduction of run-off and increased soil water storage/ permeability (Stovin et al. 2008, Day et al. 2010, Vico et al. 2014). Vegetation contributes to pollutant absorption and deposition (Tiwary et al. 2009, Tallis et al. 2011, Salmond et al. 2016).

Vegetation influences the momentum flux by exerting drag on the mean wind flow (Finnigan 2000, Guan et al. 2003, Krayenhoff et al. 2015, Giometto et al. 2017). At critical aerodynamic porosities (*P*_*3D*_) this drag can be as significant as solid structures of the same shape (Hagen and Skidmore 1971, Mayhead 1973, Grant and Nickling 1998, Guan et al. 2000, 2003, Rudnicki et al. 2004, Vollsinger et al. 2005, Koizumi et al. 2010, Kent et al. 2017a). Vegetation therefore influences the spatially-averaged mean and turbulent characteristics of the flow in urban areas (Krayenhoff et al. 2015), having implications for in-canopy flow (Salmond et al. 2013), as well as the exchange between in- and above-canopy air masses (Gromke and Ruck 2009, Vos et al. 2013).

The influence of a defined surface area upon fluxes of momentum can be indicated using the aerodynamic parameters of the zero-plane displacement (*z*_*d*_) and aerodynamic roughness length (*z*_*0*_), which are directly related to surface characteristics. Several methods exist to determine these, including algorithms based upon surface form (morphometric methods) or observations (anemometric methods). The presence of all roughness elements is inherently included in anemometric methods, but until recently morphometric methods did not consider both vegetation and buildings in combination. However, Kent et al. (2017a) develop the widely-used Macdonald et al. (1998) (hereafter *Mac*) morphometric method to include vegetation, which also applies to the Kanda et al. (2013) (*Kan*) extension of the *Mac* method.

The objectives of this paper are to use observations at two vegetated urban sites to investigate: (i) the seasonal variability in *z*_*d*_ and *z*_*0*_ with the seasonal change of tree phenology, (ii) Kent et al.’s (2017a) parameterisation of vegetation in the morphometric methods and (iii) the implications of considering vegetation for accurate wind-speed estimation. The interdependence of *z*_*d*_ and *z*_*0*_ means that a single value for each parameter cannot be treated as the ‘truth’. Therefore, the analysis provides a comparison between the magnitude and directional variability of roughness parameters determined from the different methods. The wind-speed estimation application provides an independent assessment of the method performance.

## Methodology

### Site description and observations

**Fig. 1:**
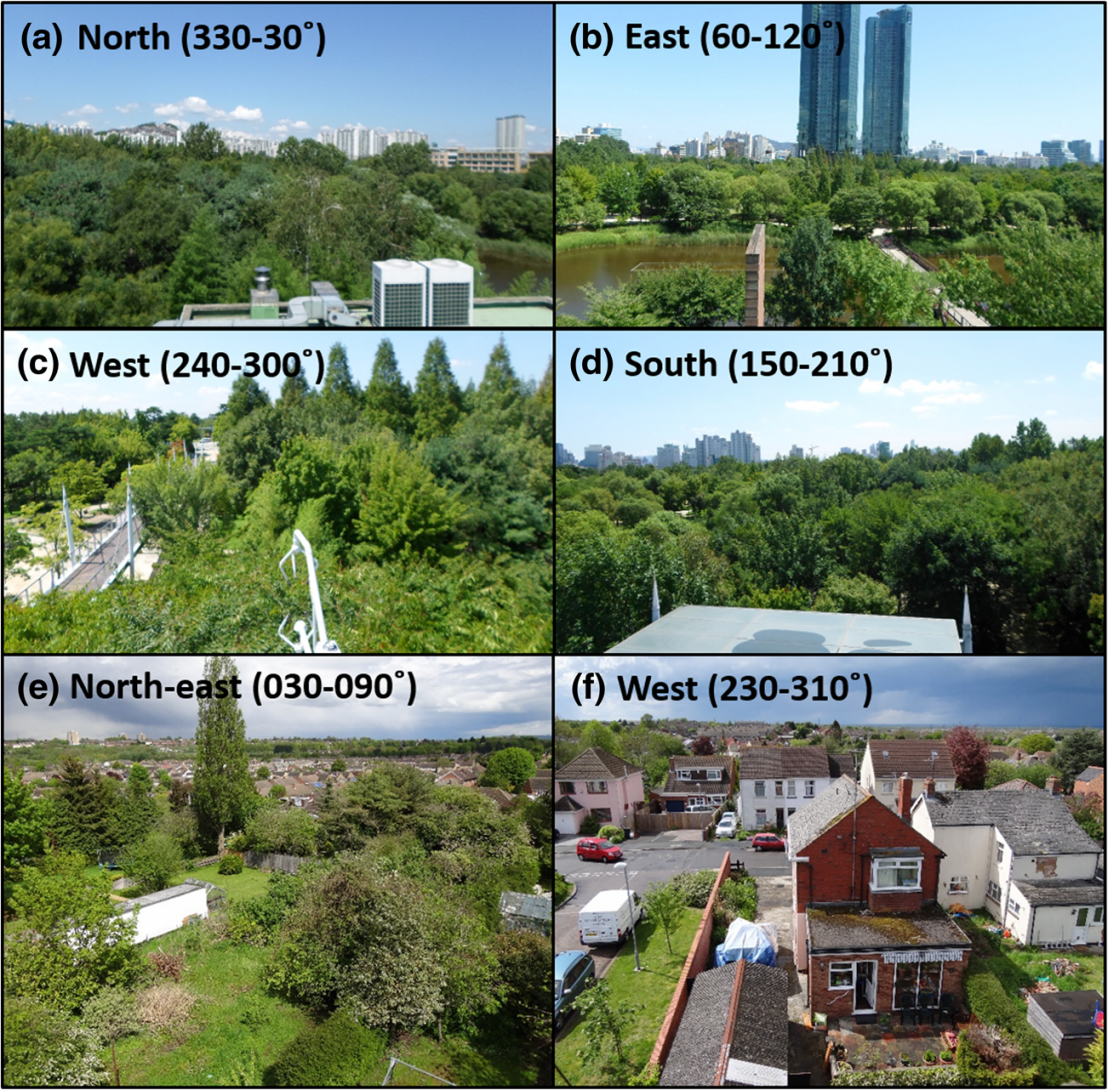
View from the: **(a-d)** Seoul Forest Park (SFP) and **(e-f)** Swindon (SWD) measurement locations, with approximate directions

Measurements from an urban park in Seoul, South Korea (Seoul Forest Park, SFP) and a suburban residential neighbourhood in Swindon, UK (SWD) are used. The obvious contrast of landscape with vegetation phenology means trees and other vegetation are expected to influence the aerodynamic properties of both areas, especially during leaf-on conditions when foliage is at relative maxima. Seoul Forest Park is the third largest park in Seoul (~116 ha), with a dominance of vegetation evident (Fig. 1a-d). The SWD site is typical of UK suburbia, with a slightly larger proportion of buildings than vegetation, but this varies with direction (e.g. Fig. 1e, f). Considerable research at the SWD site means anthropogenic and biogenic controls of energy, water and carbon fluxes and their temporal variability are well understood (Ward et al. 2013, 2014, 2015a, 2015b, 2015c). In addition, the site has been used during development of the Surface Urban Energy and Water Balance Scheme (SUEWS) (Ward et al. 2016). However, in-depth aerodynamic parameter analysis has not been performed at either the SFP or SWD site.

At each site, fast-response observations of temperature, wind velocity (*u* – horizontal, *v* – transverse and *w* – vertical components), CO_2_ and H_2_O are processed into 30-min averages (Table 1).

**Table 1:** Site observation meta-data. Heights are metres above ground level

Site: Lat, Lon (WGS84)	Local climate zone (LCZ)*	Observation period	Measurement height. Mounting.	Instrumentation	Data processing
Seoul Forest Park (SFP) 37° 32′ 40.7″N 127° 2′ 16.4″E	Scattered trees (type B): predominantly mixed forest (Pine, Ginkgo, Zelkova trees), pond and turf grass. Becoming dense trees (type A) within 300 m radius.	31 May 2013–3 June 2015	12.2 m. 230^o^ orientation on 3.8 m tripod atop of 8.4 m duplex building.	CSAT3 Sonic Anemometer; EC155 closed-path gas analyser (Campbell Scientific, USA)	Raw 10 Hz data processed to 30-min averages with spike detection (Papale et al. 2006; Hong et al. 2009), night-time correction (Aubinet et al. 2000) and double rotation of the wind components, aligning the wind field to the *u* direction (McMillen 1988, Kaimal and Finnigan 1994).
Swindon (SWD) 51° 35′ 4.6″N 1° 47′ 53.2″W	Open low-rise: well-spaced low-rise residential buildings and abundant pervious land cover	9 May 2011–30 April 2013	12.5 m. Pneumatic mast.	R3 Sonic Anemometer (Gill Instruments, Lymington, UK); LI-7500 open-path gas analyser (LI-COR Biosciences, Lincoln, USA)	Raw 20 Hz data processed to 30-min averages using EddyPro Advanced (v5–00, LI-COR), which includes de-spiking, double coordinate rotation, humidity correction of sonic temperature and high- and low-frequency spectral corrections (Moncrieff et al. 1997).

*(Stewart and Oke 2012)

### Surface elevation database and differentiation between buildings and vegetation

At both sites, 1-m horizontal resolution digital surface (DSM, ground height + surface features) and digital terrain (DTM, ground height only) models are analysed (Table 2). The high resolution and accuracy of these data, allow intricacies of surface roughness (e.g. roof pitch) to be resolved. After subtraction of the DTM from the DSM to provide a roughness element surface model (RESM), pixels <2 m high are removed (i.e. street furniture and temporary obstacles, such as vehicles). This retains roughness elements which are most appropriate for application of the morphometric methods. Building and vegetation pixels are differentiated by three techniques.

**Table 2:** Source and accuracy of surface elevation databases used at the Seoul Forest Park (SFP) and Swindon (SWD) measurement sites

Site	Elevation data source	Horizontal resolution (m)	Accuracy: horizontal, vertical (m)
SFP	National Geographic Information Institute	1	0.15, 0.10
SWD	Environment agency (UK) data archive	1	0.40, 0.15

**Fig. 2:**
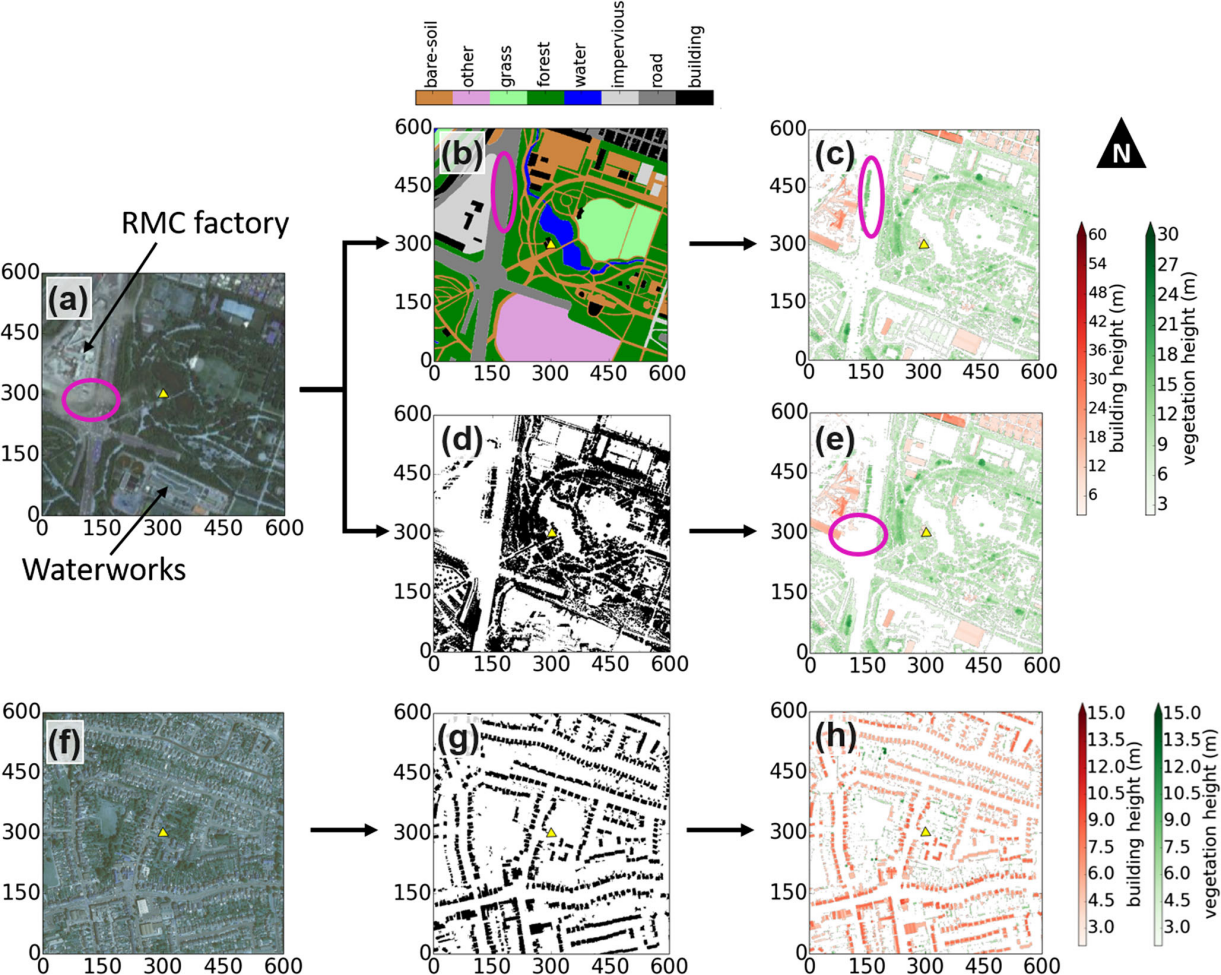
Classification of buildings and vegetation for the **(a**-**e)** Seoul Forest Park and **(f**-**h)** Swindon site (yellow triangles) surroundings: **(a, f)** aerial photograph; **(b)** manual land cover classification; **(c)** building digital surface model (BSM, red) and canopy digital surface model (CDSM, green) from manual technique; **(d)** vegetation mask from analysis of RGB colour bands in **(a)**; **(e)** BSM and CDSM from automated technique; **(g)** building footprints; and, **(h)** BSM and CDSM using building footprint mask. Magenta circles are referred to in text. Map units are metres. Data sources: aerial imagery – Seoul city aerial image service centre, Digimap 2017; elevation data – see Table 2; building footprints – Ordnance Survey 2014

For the SFP site, initial source area calculations (using the Kormann and Meixner (2001) and Kljun et al. (2015) models) indicate the measurements are consistently influenced by an area within 300 m of the sensor. The area within this radius is classified using a manual and automated technique. The manual technique entails classification of aerial photography (Fig. 2a) into: building, road, impervious, water, forest, grass, bare soil and other (unclassified, but with few roughness elements) (Fig. 2b), with the RESM data overlain to check for inconsistencies. This manual method has some limitations, for example, although buildings (predominantly rectangular with sharp boarders) are mostly captured, those within a waterworks (south of the SFP site) and in a ready mixed concrete (RMC) factory (north-west) are misclassified (Fig. 2b). Additionally, considerable vegetation is missed, especially at land cover interfaces (e.g. along roadsides and bare soil paths, Fig. 2b and c, magenta circles). After re-classification, a surface model of building (BSM) and vegetation canopy (CDSM) heights is created (Fig. 2c).

The automated separation of buildings and vegetation, uses the RGB colour band of aerial imagery, as vegetation tends to be darker (i.e. lower end of the saturation spectrum) for all colour bands. If higher saturation pixels are removed, a binary mask representing pixels which are likely vegetation can be retained (e.g. Fig. 2d) (Crawford et al. 2016). Clouds in the imagery causes some vegetation to be uncaptured by the mask (cf. Fig. 2a and e, magenta circle). A dark to lighter pixel transition on the edge of vegetation means the mask may be smaller than vegetation’s true extent. Therefore, the binary mask and RESM are combined and a filtering algorithm flags pixels as vegetation if they are within ±3 m of another pixel in the binary mask. Pixels not flagged are either buildings or other urban furniture (e.g. cars, street lamps etc.). After removing pixel heights < 2 m, a final CDSM and BSM product is generated (Fig. 2e).

Although the manual (after re-classification) and automated CDSM and BSM products are almost identical (cf. Fig. 2c and e), the latter method is more practical. The remainder of this work uses a combined dataset from both procedures.

At the SWD site, the abundance of vegetation and proximity of built structures makes accurate manual classification difficult. Additionally, the automated technique frequently misclassifies building pixels as vegetation because of the dark roofs and excessive shading (e.g. Fig. 2f). Therefore, a building footprint dataset (OS MasterMap® Topography Layer – Building Height Attribute, Ordnance Survey 2014) (Fig. 2g) was overlain upon the RESM to create the BSM (Fig. 2h, red). The remaining pixels were classed as ‘potential’ vegetation pixels, with isolated pixels removed if fewer than 6 of the 8 surrounding pixels were not ‘potential’ vegetation (Goodwin et al. 2009, Lindberg and Grimmond 2011). The remaining pixels were stored as a CDSM (Fig. 2h, green).

### Calculation of aerodynamic roughness parameters

Two anemometric methods are used to determine *z*_*d*_: the temperature variance (TVM, Rotach 1994) and wind variance (WVM, Toda and Sugita 2003) methods. The TVM and WVM are based upon the relation between the non-dimensional standard deviation of temperature or vertical wind and stability parameter in the surface layer, during unstable conditions (Wyngaard et al. 1971, Tillman 1972):

1 $$ {\phi}_T=\frac{\sigma_T}{T_{\ast }}=-{C}_1{\left({C}_2-\frac{z-{z}_d}{L}\right)}^{-\frac{1}{3}} $$

2 $$ {\phi}_w=\frac{\sigma_w}{u_{\ast }}={C}_3{\left(1-{C}_4\left[\frac{z-{z}_d}{L}\right]\right)}^{\frac{1}{3}} $$

where *σ*_*T*_ and *σ*_*w*_ are the standard deviation of temperature and vertical wind velocity respectively, *T*_∗_ is the temperature scale, ${T}_{\ast }=-\left(\overline{w^{\prime }{T}^{\prime }}\right)/{u}_{\ast }$ (with *T* the temperature, *w* the vertical wind velocity, *u*_∗_ friction velocity, the overbar representing a mean value and prime indicating deviation from the mean), *L* is the Obukhov length, $L=\frac{\overline{T}{u_{\ast}}^2}{\kappa g{T}_{\ast }}$ (with *g* the gravitational acceleration and *κ* von Karman’s constant = 0.4, Högström 1996) and *C*_*1*_ – *C*_*4*_ are constants.

The TVM and WVM are amongst the few methods that permit roughness parameters to be derived from single-level turbulence measurements. However, the methods rely on Monin-Obukhov similarity theory and that the resulting flux gradient relations used by the TVM and WVM (Eq. 1 and 2) apply in urban areas (see Roth and Oke 1995). Therefore, the applicability of the similarity relations used by the methods is assessed at both sites during this work. Although the similarity relations are expected to hold where flow is free from roughness-element wakes (i.e. within the inertial sublayer), the TVM is specifically developed to determine *z*_*d*_ from measurement locations which may be distorted by local roughness-element wakes (i.e. within the roughness sublayer) (Rotach 1994). Previous analysis indicates results from the WVM are appropriate in similar heterogeneous locations (Toda and Sugita 2003, Kent et al. 2017b).

The constants (*C*_*1*_ to *C*_*4*_) are derived from observations when *z*_*d*_ is assumed negligible. Although the constants vary (e.g. Sorbjan 1989, Hsieh et al. 1996, Choi et al. 2004), the *z*_*d*_ from the temperature and wind variance methods was found to be relatively insensitive to the range in a dense urban area (Kent et al. 2017b). To assess the effect of constant choice on the final solution to *z*_*d*_ the methods are applied with a range of constants (Table 3). Note, if constants are fit to the observations at a site an *a priori* assumption of *z*_*d*_ is required and therefore the *z*_*d*_ retrieved is not useful (Kent et al. 2017b).

**Table 3:** Constants (*C*_*1*_ – *C*_*4*_) for application of the temperature variance (TVM) and wind variance (WVM) anemometric methods (Eq. 1 and 2). For all observations, extensive flat homogeneous terrain is reported. Kaimal and Finnigan (1994) and Toda and Sugita (2003) are after synthesis of coefficients from various studies. At the SWD site, the Choi et al. (2004) constants are not applied, as they predict the scaled *σ*_*T*_ and *σ*_*w*_ to be much larger and smaller than observations, respectively, meaning *z*_*d*_ solutions are consistently zero

Reference	TVM		WVM	
	*C*_*1*_	*C*_*2*_	*C*_*3*_	*C*_*4*_
Tillman (1972)	0.95	0.050	–	–
Panofsky et al. (1977)	–	–	1.30	3.00
De Bruin et al. (1993)*	0.95	0.035	–	–
Kustas et al. (1994)	1.1	0.085	–	–
Kaimal and Finnigan (1994)*	1.05	0.040	1.25	3.00
Toda and Sugita (2003)	0.99	0.060	1.25	3.00
Choi et al. (2004)*	1.14	0.030	1.12	2.80

*constants obtained from ${\sigma}_T/{T}_{\ast }={C}_1{\left(1-{C}_2\left[\left(z-{z}_d\right)/L\right]\right)}^{-\frac{1}{3}}$

The right-hand sides of Eq. 1 and 2 are estimated by increasing *z*_*d*_ from zero to twice the measurement height (*z*_*m*_) in 0.1 m increments (producing *ϕ*_*est*_). The *z*_*d*_ is the value which minimises the root-mean-square error (RMSE) between *ϕ*_*est*_ and the observed value (*ϕ*_*obs*_) of *σ*_*T*_/*T*_∗_ or *σ*_*w*_/*u*_∗_ (for the TVM and WVM, respectively). As calculations are undertaken for unstable conditions (0.05 ≤ −*z’/L* ≤ 6.2, Roth 2000; *z’* = *z*_*m*_ – *z*_*d*_) an initial *z*_*d*_ for stability definition is required. Thus, the methods are applied to 10^o^ wind sectors around the sites with: (i) the *z*_*d*_ for stability definition varied from 0 to 10 m in 2-m increments (a larger initial *z*_*d*_ provides insufficient data to apply the methods); and (ii) different constants (i.e. Table 3).

If measurements are free from roughness-element wakes (i.e. within the inertial sublayer), the ‘eddy-covariance (EC) method’ can be used to determine *z*_*0*_, which is a rearrangement of the logarithmic wind law:

3 $$ {z}_0=\left(z-{z}_d\right)\exp \left(-\frac{{\overline{U}}_z\kappa }{u_{\ast }}\right) $$

where the average wind speed (${\overline{U}}_z$) and *u*_∗_ are determined from observations at *z*_*m*_. For each 30-min period of observations, *z*_*d*_ from both the temperature and wind variance methods are used, providing two *z*_*0*_ solutions. The EC method, applicable under neutral conditions (|*z’/L*| ≤ 0.05), requires at least 20 observations to determine *z*_*0*_ for a directional sector (Beljaars 1987, Grimmond et al. 1998). Additionally, only ${\overline{U}}_z$ > 1 m s^−1^ are analysed to ensure sufficient mechanical turbulence (Liu et al. 2009). Stability corrections may be used to apply the EC method outside of neutral conditions. However, these corrections are based upon empirical fits to observed data and vary across studies (Högström 1996). To avoid additional sources of uncertainty only neutral conditions are considered here.

As the SFP site results indicate *z*_*d*_ is similar to (or greater than) *z*_*m*_, the EC method to determine *z*_*0*_ is therefore unusable (and not applied). For both northern-hemisphere sites, leaf-off periods are selected as the (core) winter months of December, January and February; and leaf-on periods are June, July and August. With little solar radiation during winter (leaf-off periods) at the SWD site there are insufficient unstable periods to determine *z*_*d*_ using the temperature and wind variance methods (and hence *z*_*0*_). Therefore, only leaf-on conditions are analysed at the SWD site.

The Macdonald et al. (1998, *Mac*) and Kanda et al. (2013, *Kan*) morphometric methods are used with a new vegetation parameterisation (Kent et al. 2017a). Following the Kent et al. (2017b) methodology, an iterative procedure is applied using the Kormann and Meixner (2001) footprint model with 30-min averaged meteorological observations. Initial rural *z*_*d*_ and *z*_*0*_ values (0.2 and 0.03 m, respectively) are used, as results are independent of these values when applying an iterative procedure (Kent et al. 2017b). Morphometric calculations are only applied to source areas which extend horizontally beyond 50 m from the measurement sensors, as smaller source areas become concentrated upon only a few roughness elements and the morphometric calculations are inappropriate.

For each 30-min observation, the source area weighted geometry is calculated for buildings and vegetation (using the BSM and CDSM). The average, maximum and standard deviation of *all* roughness-element heights (*H*_*av*_, *H*_*max*_ and *σ*_*H*_, respectively) are determined. The plan area index (*λ*_*p*_) of roughness elements is:

4 $$ {\lambda}_p=\frac{W_{p,b}+{W}_{p,v}\left(1-{P}_{3D}\right)}{W_{AT}} $$

where *W*_*p,b*_ and *W*_*p,v*_ are the sums of weighted pixels in the source area of buildings and vegetation, respectively, *W*_*AT*_ is the total sum of weights and *P*_*3D*_ is the aerodynamic porosity of vegetation. The weighted frontal area of buildings and vegetation is determined separately (*W*_*f,b*_ and *W*_*f,v*_), treating vegetation as non-porous.

Including vegetation, the *Mac* method becomes (Kent et al. 2017a):

5 $$ {Mac_z}_d=\left[1+{\alpha}^{-{\uplambda}_p}\left({\uplambda}_p-1\right)\right]{H}_{av} $$

6 $$ {Mac}_{z_0}=\left(\left(1-\frac{z_d}{H_{av}}\right)\exp \left[-{\left(0.5\beta \frac{C_{Db}}{k^2}\left(1-\frac{z_d}{H_{av}}\right)\frac{\left\{{W}_{f,b}+{W}_{f,v}\left({P}_v\right)\right\}}{W_{AT}}\right)}^{-0.5}\right]\right){H}_{av} $$

where *C*_*Db*_ = 1.2 is the drag coefficient for buildings and *α* = 4.43 and *β* = 1.0 are empirical constants for staggered arrays fit to the wind tunnel data of Hall et al. (1996). *P*_*v*_ is the ratio between the drag coefficient for vegetation with varying *P*_*3D*_ and buildings (Kent et al. 2017a):

7 $$ {P}_v=\frac{-1.251{P_{3D}}^2+0.489{P}_{3D}+0.803}{C_{Db}} $$

derived from experiments with 0 ≤ *P*_*3D*_ ≤ 0.85 (Guan et al. 2000). The *Kan* method is a development of the *Mac* method, incorporating roughness-element height variability (Kanda et al. 2013):

8 $$ {Kan}_{z_d=}\left[{}_{C_0}{X}^2+\left({a}_0{\lambda}_p^{b_0}{-}_{C_0}\right)X\right]{H}_{\mathrm{max}},X=\frac{\sigma_H+{H}_{av}}{H_{\mathrm{max}}} $$

and

9 $$ {Kan}_{z_0=}\left({b}_1{Y}^2+{c}_1Y+{a}_1\right){Mac}_{z_0},Y=\frac{\lambda_p{\sigma}_H}{H_{av}} $$

where 0 ≤ *X* ≤ 1, 0 ≤ *Y* and *a*_*0*_, *b*_*0*_, *c*_*0*_, *a*_*1*_, *b*_*1*_ and *c*_*1*_, are regressed constants of 1.29, 0.36, −0.17, 0.71, 20.21 and −0.77.

The methods are applicable to any combination of buildings and vegetation, with vegetation phenology and associated drag characteristics being optimisable (through *P*_*3D*_). With this information being scarce, and the predominance of deciduous vegetation at both sites it is assumed that all vegetation has a leaf-on porosity of 20% and leaf-off porosity of 60% (i.e. *P*_*3D*_ = 0.2 and *P*_*3D*_ = 0.6, respectively, Heisler 1984; Heisler and DeWalle 1988, Grimmond and Oke 1999). During leaf-on and leaf-off transition an intermediate porosity may be used (e.g. *P*_*3D*_ = 0.4). However, the rapid transition at both sites (< 30 days) means there is insufficient data to investigate the transition periods here.

Determination of source-area weighted aerodynamic parameters using the morphometric methods (including vegetation) are implemented into the Urban Multi-scale Environmental Predictor (UMEP, http://www.urban-climate.net/umep/UMEP) climate service plugin for the open source software QGIS (Lindberg et al. 2018).

## Results

### Impact of roughness elements on observational data

**Fig. 3:**
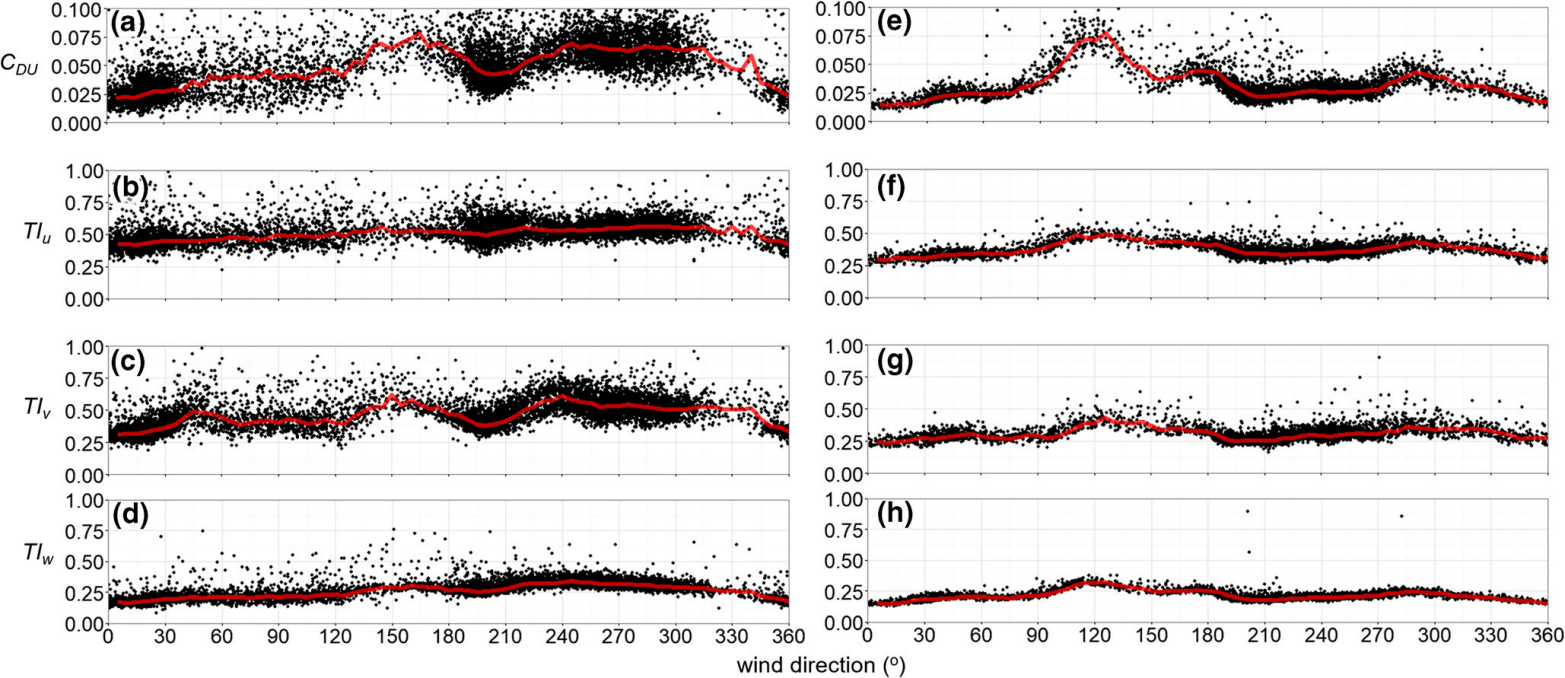
All 30-min observations during neutral conditions (black dots) at the **(a-d)** Seoul Forest Park (SFP) and **(e-h)** Swindon (SWD) sites: **(a, e)** Aerodynamic drag coefficient (*C*_*DU*_ = ${\left({u}_{\ast }/{\overline{U}}_z\right)}^2$) and turbulence intensities in the **(b, f)** longitudinal (*TI*_*u*_ *= σ*_*u*_*/*${\overline{U}}_z$), **(c, g)** transverse (*TI*_*v*_ *= σ*_*v*_*/*${\overline{U}}_z$) and **(d, h)** vertical (*TI*_*w*_ *= σ*_*w*_*/*${\overline{U}}_z$) wind directions. Neutral conditions are |(*z*_*m*_ *– z*_*d*_)*/L*| ≤ 0.05 (*L* is Obukhov length, *z*_*m*_ = 12.2 m at SFP and 12. 5 m at SWD, *z*_*d*_ is assumed as *H*_*av*_ = 8 m at SFP and 4.5 m at SWD). Red line is the median for each 5^o^ wind direction. ${\overline{U}}_z$ is the average wind speed, *u*_∗_ is the friction velocity and σ_*u*_, *σ*_*v*_, *σ*_*w*_ are the standard deviations of the longitudinal, transverse and vertical velocity components of the wind

To assess the disturbance to measurements from nearby roughness elements the turbulence data are inspected (Fig. 3). At the SFP site, the data are more variable due to the proximity to roughness elements (measurements are at 1.6*H*_*av*_ of all roughness elements in the 300-m radius) (Fig. 3a-d). In some directions *z*_*m*_ is similar to *H*_*av*_ (N, SW, W, NW, Table 4b), and *H*_*max*_ is always larger than *z*_*m*_. Therefore, the measurements are probably within the roughness sublayer (RSL) and *z*_*d*_ is often larger than *z*_*m*_. A peak in the aerodynamic drag coefficient and transverse turbulence intensity between 130^o^ – 180^o^ is likely caused by the rear sides of the sensor (Fig. 3a, c). In addition, there is a larger proportion of drag between 210^o^ – 330^o^ where taller roughness elements are located (Fig. 3a).

**Table 4:** Characteristics within a 300-m radius of the Seoul Forest Park site by direction (45^o^ sectors, ±22.5^o^ of the stated direction): **(a)** Land cover and **(b)** Geometry of roughness elements > 2 m. ‘Other’ land cover is predominantly comprised of a water works (with few roughness elements > 2 m), which cannot be classified from aerial imagery

(a) Land cover (%)										
Direction	Building	Impervious (road)	Impervious (non-road)	Water	Vegetation	Grass	Other	Bare-soil		
N	12	7	0	7	40	0	0	34		
NE	18	4	5	3	49	8	0	13		
E	1	0	0	4	54	33	0	8		
SE	8	1	3	4	68	0	2	14		
S	5	6	0	0	48	0	39	2		
SW	2	25	1	0	54	0	8	10		
W	16	29	20	0	33	0	0	2		
NW	16	33	15	0	30	2	0	4		
(b) Geometry										
Direction	Buildings					Vegetation				
	*H*_*av*_	*H*_*max*_	*σ*_*H*_	*λ*_*p*_	*λ*_*f*_	*H*_*av*_	*H*_*max*_	*σ*_*H*_	*λ*_*p*_	*λ*_*f*_
N	12.65	33.4	8.43	0.11	0.07	7.5	28.06	3.7	0.33	0.38
NE	7.38	16.53	2.23	0.13	0.02	7.63	22.16	2.81	0.44	0.29
E	6.46	11.57	2.80	0.00	0.00	7.56	21.91	2.57	0.43	0.34
SE	6.68	12.83	1.92	0.05	0.03	6.98	19.43	2.73	0.47	0.34
S	6.16	8.82	1.06	0.05	0.01	5.76	17.51	3.17	0.41	0.31
SW	11.31	16.93	4.75	0.02	0.01	7.64	22.86	3.39	0.40	0.25
W	11.04	27.67	6.93	0.15	0.12	8.14	18.84	3.37	0.25	0.24
NW	8.29	26.45	5.32	0.08	0.09	9.47	20.74	3.62	0.20	0.20

*Geometry abbreviations*: *H*_*av*_ – average height, *H*_*max*_ – maximum height, *σ*_*H*_ – standard deviation of heights, *λ*_*p*_ – plan area index, *λ*_*f*_ –frontal area index

Although the TVM and WVM methods have been demonstrated to be appropriate in the RSL, the calculation of source areas is more uncertain (e.g. Baldocchi 1997, Rannik et al. 2000, Sogachev and Lloyd 2004, Vesala et al. 2008, Leclerc and Foken 2014). However, Fig. 3a-d demonstrates there is still some homogeneity to the flow and to characterise the local roughness, the Kormann and Meixner (2001) footprint model is applied at a height likely greater than the RSL (2.5*H*_*av*_ = 20 m).

At the SWD site, the measurement height is approximately 2.8*H*_*av*_ based on the measurement source area. Combined with a lack of disturbance to turbulence data for most directions (Fig. 3e-h), this indicates measurements are taken within the inertial sublayer (ISL), where it is most appropriate to apply the anemometric methods and source area calculations. Peaks in turbulence data between 100^o^ and 140^o^ and at approximately 180^o^ and 280^o^ (Fig. 3e-h) are likely caused by houses with maximum heights of up to 8 m – 10 m in these directions (within 25 m of the sensor).

### Land cover and geometry surrounding the sites

Source areas indicate the likely surface influencing turbulent fluxes measured at a point (Schmid and Oke 1990, Schmid 1997, Leclerc and Foken 2014) and can therefore be used to characterise site surroundings, with varying certainty (Heidbach et al. 2017). Source area characterisation is performed for the SWD site. However, at the SFP site, the uncertainty in the calculated source areas and the large *z*_*d*_ compared to *z*_*m*_ means 45^o^ direction sectors are used.

Aerodynamic characteristics are expected to be dominated by the tall and abundant vegetation at the SFP site, rather than by the sparse buildings. The average vegetation height (*H*_*av,v*_) ranges between 5 and 10 m and with a maximum (*H*_*max,v*_) of ≥ 17.5 m in all directions it is over double *H*_*av*_. The plan and frontal area indexes of vegetated roughness elements (*λ*_*p,v*_ and *λ*_*f,v*_, respectively) are consistently > 0.3, whereas buildings have plan and frontal area indexes (*λ*_*p,b*_ and *λ*_*f,b*_) consistently < 0.1 (Table 4b). An exception is to the north and west where the built fraction increases to close to 20% due to the RMC factory (Fig. 2a).

**Fig. 4:**
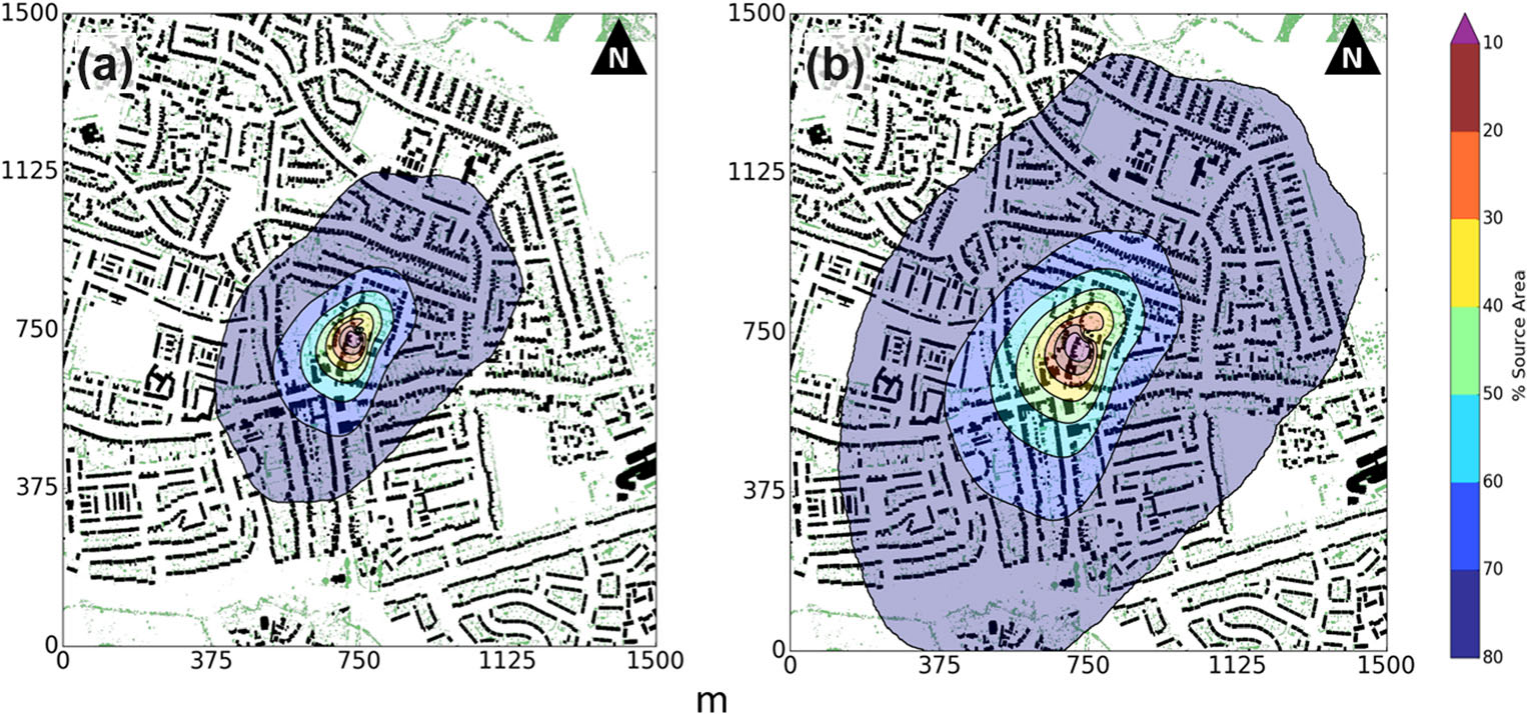
Source area climatology of the Swindon site (SWD) modelled using the Kormann and Meixner (2001) source area model for the months of June, July and August (Leaf-on) in 2011 and 2012. Source areas are modelled using aerodynamic parameters from the **(a)** Kanda et al. (2013) and **(b)** Macdonald et al. (1998) morphometric methods. The 80% cumulative source area weights for each 30-min average of observations are integrated and normalised by the sum of all weightings (*n* = 8787). Source areas overlain upon buildings (black) and vegetation (green) > 2 m

Source area size varies with the model and parameters used (e.g. Leclerc and Foken 2014, Heidbach et al. 2017), as demonstrated with aerodynamic parameters (*z*_*d*_ and *z*_*0*_) from the *Kan* and *Mac* morphometric methods (Fig. 4). Independent of morphometric method, the SWD site source areas include residential housing, back gardens and impervious driveways. The source area climatology is biased towards the predominant south-westerly wind direction, where it also extends further upwind because of the greater wind speeds in this direction.

The *Mac* method source areas are larger than the *Kan* method (average upwind extents of 700 m and 400 m, respectively) due to the difference in aerodynamic parameters determined with each method. The peak flux footprint is 50 m upwind for the *Mac* method and 25 m upwind for *Kan* (Fig. 4). Although these differences impact the surface geometry and land cover determined within the source area, the consistent fetch at the SWD site means the parameters determined by the *Mac* and *Kan* source areas are remarkably similar (Table 5). The source area has 11% built and 2% vegetated roughness elements, with low-level vegetation (i.e. small shrubs), grass, impervious materials (e.g. roads or driveways) and soil forming the remainder. The latter have a comparatively small impact on the aerodynamic roughness parameters at the neighbourhood scale. The average height of vegetation is smaller than buildings (~3.5 m and 4.7 m, respectively), but the maximum tree height (up to 15 m) is slightly larger than buildings (up to 12 m). To the north-east (030^o^ – 090^o^), trees are tallest and most abundant, whilst vegetation is least to the south-west (Fig. 2f, 210 – 240^o^).

**Table 5:** Roughness-element characteristics in the source areas modelled during leaf-on conditions at SWD using the **(a)** Kanda et al. (2013, *Kan*) and **(b)** Macdonald et al. (1998, *Mac*) morphometric methods (Fig. 4) for all observations (*n* = 8787) and in the specified north-easterly (NE) (*n* = 911) and south-westerly (SW) (*n* = 2045) directions

Direction	Buildings						Vegetation					
	*H*_*av*_	*H*_*max*_	*σ*_*H*_	*λ*_*p*_	*λ*_*f*_	Cover (%)	*H*_*av*_	*H*_*max*_	*σ*_*H*_	*λ*_*p*_	*λ*_*f*_	Cover (%)
(a) Kan												
All	4.74	10.05	1.59	0.24	0.13	11	3.49	12.08	1.58	0.05	0.11	2
NE (030°-090°)	3.92	9.80	1.31	0.15	0.06	6	3.88	13.03	1.70	0.07	0.14	4
SW (210°-240°)	4.52	10.40	1.66	0.30	0.15	14	3.35	12.33	1.59	0.04	0.10	2
(b) Mac												
All	4.67	11.96	1.65	0.24	0.12	11	3.51	14.46	1.76	0.05	0.10	2
NE (030°-090°)	3.95	12.03	1.41	0.17	0.07	7	3.82	14.79	1.73	0.07	0.13	3
SW (210°-240°)	4.62	12.01	1.73	0.30	0.14	13	3.39	13.76	1.61	0.04	0.09	2

*Geometry abbreviations*: *H*_*av*_ average height, *H*_*max*_ maximum height, *σ*_*H*_ standard deviation of heights, *λ*_*p*_ plan area index, *λ*_*f*_ frontal area index. Cover is the weighted percentage of pixels in the source area which are in the BSM for buildings and CDSM for vegetation

### Aerodynamic parameters

#### Seoul Forest Park (SFP)

**Fig. 5:**
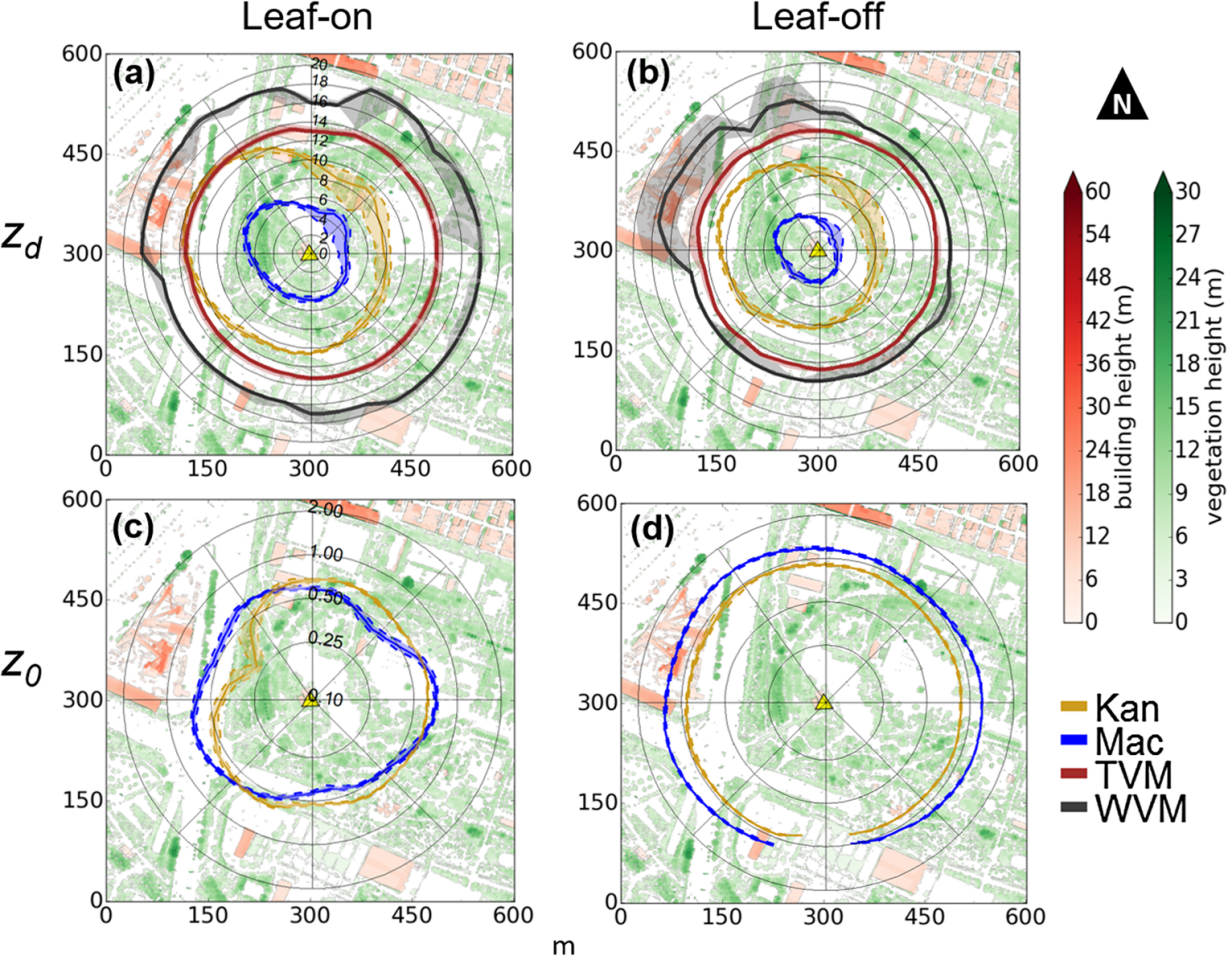
Median aerodynamic parameters determined for 10^o^ wind sectors surrounding Seoul Forest Park site (SFP). Seasons (columns) are: leaf-on (June, July, August) and leaf-off (December, January, February). *Anemometric methods:* TVM (temperature variance, Rotach 1994); WVM (wind variance, Toda and Sugita 2003), with the range of solutions (shading) provided from varying constants used during application of the methods and initial *z*_*d*_ used to define stability. *Morphometric methods*: *Kan* (Kanda et al. 2013); *Mac* (Macdonald et al. 1998) applied using the Kormann and Meixner (2001) footprint model for all 30-min observations, with 10th/ 90th percentile for each 10^o^ sector shaded. *z*_*d*_ is for unstable conditions (6.2 ≤ *z’/L* ≤ 0.05, with *z’* = *z*_*m*_ − *z*_*d*_ and *L* the Obukhov length) and *z*_*0*_ is for neutral conditions (|*z’/L*| ≤ 0.05). For morphometric method stability definition, *z*_*d*_ in *z’/L* is determined by the respective morphometric method. Insufficient neutral conditions for southerly winds during leaf-off vegetation state means no morphometric *z*_*0*_ is reported here

At the SFP site, the anemometrically determined *z*_*d*_ is relatively insensitive to both the ‘universal’ constants (Table 3) and initial *z*_*d*_ used to define stability. Both consistently cause a maximum variability of < 1 m for any 10^o^ wind sector (Fig. 5a and b, shading around grey and brown lines) which corresponds to < 10% of the median *z*_*d*_. Despite the proximity of measurements to roughness elements, the maximum RMSE between *ϕ*_*est*_ and *ϕ*_*obs*_ for the wind variance method is 0.4, which is similar to observations which are higher above roughness elements in other urban areas (Kent et al. 2017a) and provides greater confidence when using the WVM. In contrast, the RMSE for the TVM is much larger (2.0), because of the proximity to roughness elements, the thermal inhomogeneity of the area (i.e., water, grasses, trees, bare-soil, and impervious surfaces, Table 4) and the dissimilarity of roughness length between momentum and heat (e.g. Owen and Thomson 1963, Zilitinkevich 1995, Voogt and Grimmond 2000, Hong et al. 2012).

Both the TVM and WVM indicate *z*_*d*_ may be larger than *z*_*m*_ (12.2 m) for all wind directions (Fig. 5a, b). Both methods have a larger *z*_*d*_ during leaf-on (Fig. 5a) than leaf-off (Fig. 5b), which is approximately 1 m larger for the TVM and 2–4 m larger for the WVM. A seasonal increase in *z*_*d*_ is also observed by Giometto et al. (2017) for a suburban neighbourhood with the larger amount of leaf-on foliage exerting greater drag upon the flow, acting to raise the centroid of the drag profile (analogous to *z*_*d*_, Jackson 1981).

The anemometric *z*_*d*_ is more variable with wind direction during leaf-on, which can be directly related to trees (and implications for *z*_*d*_). For example, *z*_*d*_ is largest between 080^o^ – 150^o^, where there is maximum vegetation cover (> 50% land cover, Table 4a), and between 270^o^ – 010^o^ where *H*_*av,v*_ is largest (Table 4b). The 6-m variation in *z*_*d*_ between 270^o^ – 360^o^ during leaf-off (Fig. 5b), is attributed to the disturbance to airflow from the RMC factory approximately 250 m upwind (Fig. 2a), and a row of trees (> 20 m) just 60 m upwind. During leaf-on, this variability is not observed because the foliage on the trees dominates airflow disturbance, causing an obvious increase in *z*_*d*_ (Fig. 5a).

Similar to the anemometric methods, both morphometric methods indicate leaf-on *z*_*d*_ is larger than leaf-off *z*_*d,*_ (Fig. 5a, b). The effect is least obvious between 000^o^ and 120^o^ due to the lake and open grassed area. However, between 120^o^ to 280^o^ the increasing height and proportion of vegetation increases both $$ {Mac}_{z_d} $$ and $$ {Kan}_{z_d} $$, with leaf-on *z*_*d*_ 1 – 3 m larger than leaf-off (Table 4b, Fig. 5). Both morphometric methods indicate maximum *z*_*d*_ and seasonal signal between 270^o^ and 330^o^, a similar direction to the anemometric methods. Here, $$ {Mac}_{z_d} $$becomes as large as 7.5 m and $$ {Kan}_{z_d}, $$with a more pronounced peak, reaches 14 m. The latter is associated with the increased vegetation plan area, *H*_*av*_, *H*_*max*_ and *σ*_*H*_ (Table 4b). As the source area rarely extends to the RMC factory (Fig. 2a), the morphometric *z*_*d*_ is primarily a function of vegetation in these directions.

Leaf-off *z*_*0*_ is typically > 0.5 m larger than leaf-on *z*_*0*_ for both morphometric methods (Fig. 5c, d), as *z*_*0*_ varies with roughness-element density. In canopies with both vegetated (Shaw and Pereira 1982, Wolfe and Nickling 1993, Raupach 1992, 1994, Nakai et al. 2008) and built (Macdonald et al. 1998, Cheng and Castro 2002, Jiang et al. 2008) roughness elements, *z*_*0*_ has been demonstrated to increase with density until a peak *λ*_*f*_ (or leaf area index), beyond which *z*_*0*_ decreases again. Therefore, the seasonal change of *z*_*0*_ is expected to be canopy dependent: *z*_*0*_ will increase with density for sparsely packed canopies, but will decrease with density in dense canopies. The SFP site is an example of the latter, where an already densely packed canopy during leaf-off conditions becomes denser during leaf-on. This effectively closes the canopy creating a smoother surface with a flow more characteristic of a skimming regime and reduction in *z*_*0*_. The comparatively sparsely packed neighbourhood site analysed by Giometto et al. (2017) is an example of the former, whereby leaf-on transition creates an effectively rougher surface with a flow more characteristic of a wake interference regime and resultant increase in *z*_*0*_.

At the SFP site, the seasonal change in ${Mac}_{z_0}$ is more obvious than ${Kan}_{z_0}$, as the former is more sensitive to *λ*_*f*_ (e.g. Kent et al. 2017b, their Fig. 1) and ${Kan}_{z_0}$ considers geometric parameters other than *λ*_*f*_ (*H*_*max*_, *σ*_*H*_ and *λ*_*p*_). ${Kan}_{z_0}$ is on average 0.2 m less than ${Mac}_{z_0}$ because the larger *z*_*d*_ determined using the *Kan* method means physically less frontal area of roughness elements exert drag upon the mean wind flow. For both morphometric methods, leaf-off *z*_*0*_ is consistent with direction due to the relative lack of foliage (Fig. 5d). However, leaf-on *z*_*0*_ is much more directionally variable and similarly to *z*_*d*_ can be directly related to vegetation geometry and cover. ${Mac}_{z_0}$ has greater directional variability because of the aforementioned sensitivity to *λ*_*f*_.

The morphometric methods were applied treating vegetation as buildings (i.e. *P*_*3D*_ = 0) and ignoring it (i.e. *P*_*3D*_ = 1), however, the dominance of vegetation in the area meant the former produced *z*_*0*_ < 0.25 m and the latter *z*_*d*_ < 5 m. Furthermore, applying the methods without the vegetation parameterisation does not produce the seasonal change demonstrated by the observations.

#### Swindon (SWD)

**Fig. 6:**
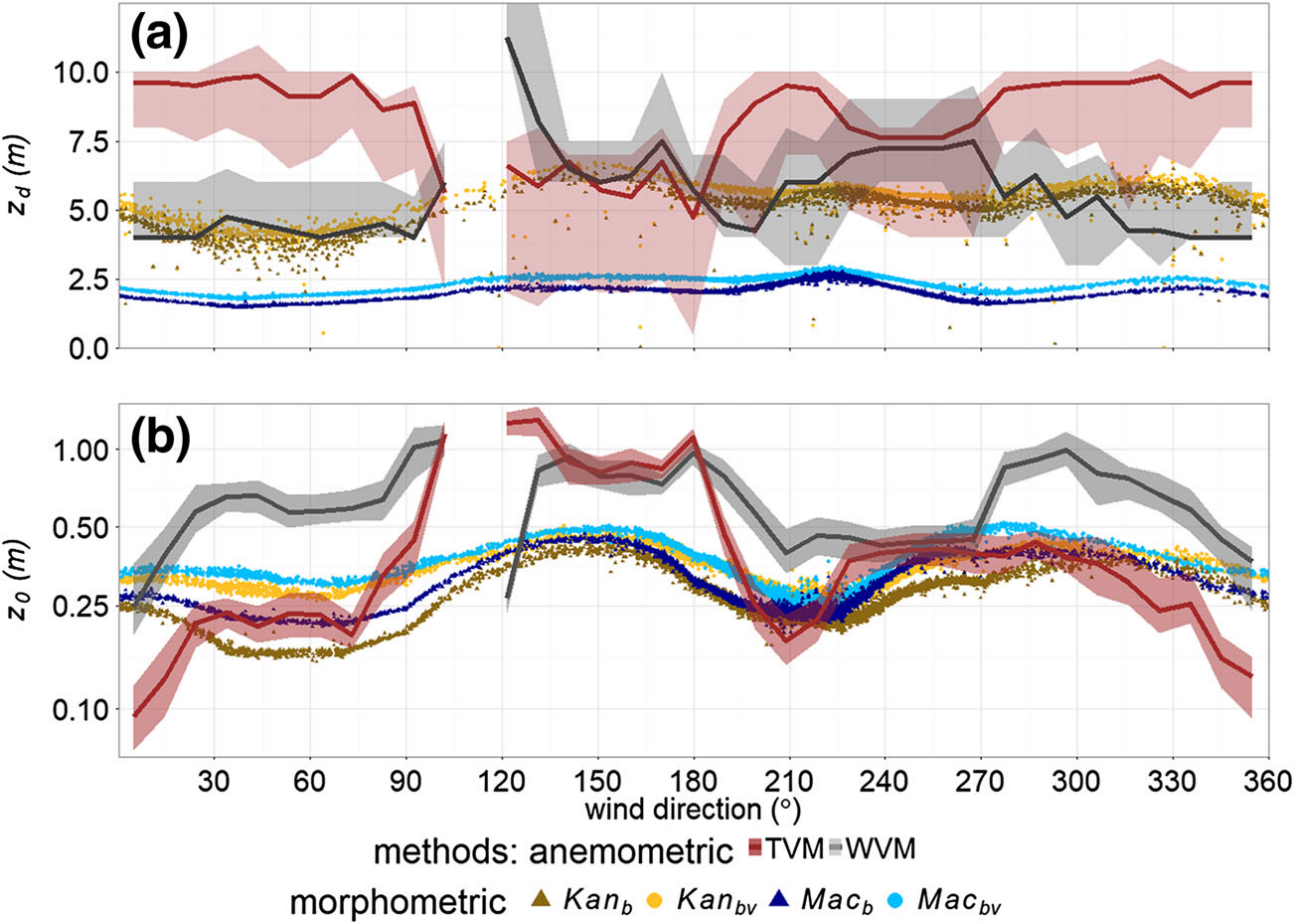
Comparison of anemometric (lines and shading) and morphometric (points) methods to determine the **(a)** zero-plane displacement (*z*_*d*_) and **(b)** aerodynamic roughness length (*z*_*0*_) (note log y axis) surrounding the Swindon site (Fig. 2f). For anemometric methods, *z*_*d*_ is the median solution of the temperature variance (TVM) and wind variance (WVM) methods, applied to 30-min observations during unstable conditions (6.2 ≤ *z’/L* ≤ 0.05, with *z’* = *z*_*m*_ *– z*_*d*_ and *L* the Obukhov length) for 10° sectors. The range (shading) represents all possible solutions by varying *z*_*d*_ used for stability definition and varying constants used in the methods. *z*_*0*_ is the median (lines) and upper and lower quartile (shaded) of the eddy-covariance method, during neutral conditions (|*z’/L*| ≤ 0.05) for each 10^o^ sector, using *z*_*d*_ from the TVM and WVM, respectively. Less than 10 observations in the 110^o^ sector means no values are reported here. The morphometric methods: *Kan* (Kanda et al. 2013); *Mac* (Macdonald et al. 1998) are for each source area during the same conditions as the anemometric methods, applied considering vegetation (subscript *bv*) and for buildings only (subscript *b*). For the morphometric method stability definition, *z*_*d*_ in *z’/L* is determined by the respective morphometric method

During leaf-on, median solutions to both the temperature and wind variance methods indicate *z*_*d*_ varies between 4 and 10 m surrounding the SWD site (Fig. 6a). However, the range of *z*_*d*_ for any 10^o^ sector is up to 5 m for the temperature variance method and up to 2.5 m for the wind variance method, corresponding to as much as 50% of median *z*_*d*_. This range is larger than previous applications of the methods (e.g. Kent et al. 2017b) and at the SFP site. The *z*_*d*_ from the temperature variance method cannot be related to surface characteristics, providing a *z*_*d*_ which is consistently close to *z*_*m*_ and up to 5 m larger than the wind variance method. The predominantly large *z*_*d*_ solutions are likely because of the thermal inhomogeneity of the area, which includes buildings, vegetated, and paved land cover. Therefore, similar to the SFP site, there is considerable variability between *ϕ*_*est*_ and *ϕ*_*obs*_ for the temperature variance method (RMSE > 0.6) and less confidence in its use.

The wind variance method indicates *z*_*d*_ is consistently between 4 and 5 m (i.e. similar to *H*_*av*_) (Fig. 6a). The directional variability of these results can be directly related to surface characteristics. Combined with the lower RMSE between *ϕ*_*est*_ and *ϕ*_*obs*_ (RMSE < 0.2), there is greater confidence in the wind variance than the temperature variance method results. Increases of *z*_*d*_ and *z*_*0*_ of up to 7.5 m and 1 m, respectively between 130^o^ – 180^o^ and 240^o^ – 280^o^ are associated with houses within 25 m of the sensor in these directions. Elsewhere, the larger *z*_*d*_ from the temperature variance method means that its associated *z*_*0*_ is consistently 0.25 m – 0.5 m less than the wind variance method (Fig. 6b).

Relative minima of both *z*_*d*_ and *z*_*0*_ occur when wind flow is aligned with the smoother road surface to the west of sites between 200^o^ – 210^o^ and 330^o^ – 360^o^ (Fig. 6a). A relative increase in *z*_*0*_ for both methods (to approximately 0.25 m and 0.75 m for the temperature and wind variance methods, respectively) in the 045^o^ direction is likely because of the taller and more abundant vegetation in the same direction. However, there is not a similar increase of *z*_*d*_.

For both *z*_*d*_ and *z*_*0*_ the morphometric methods have less directional variability than the anemometric methods because of the similarity in geometry surrounding the SWD site. When vegetation is considered ${Kan}_{z_d}$ ranges between 2.5 m and 7 m and is therefore approximately 0.5–1.5*H*_*av*_ (Fig. 6a, *Kan*_*bv*_). In comparison ${Mac}_{z_d}$ is consistently half of this, ranging between 0.25–0.75*H*_*av*_ (Fig. 6a, *Mac*_*bv*_). The *z*_*d*_ determined by the morphometric methods is more similar to the wind variance method (than the temperature variance), especially for ${Kan}_{z_d}$, which has an average difference of 0.2 m. The methods indicate relative minima and maxima of *z*_*d*_ in similar directions. A relative reduction between 030^o^ – 090^o^ occurs where fewer buildings are located (Table 5), whilst an increase between 130^o^ – 180^o^ is associated with the taller buildings close to the sensor and larger *H*_*av*_ (~5 m).

For aerodynamic roughness length, incorporating vegetation in the morphometric calculations means *z*_*0*_ ranges between 0.2 m and 0.5 m. The similarity of *z*_*0*_ between the methods is because the frontal area index ranges between 0.15 and 0.2, a region that the methods indicate similar *z*_*0*_ (Kent et al. 2017b their Fig. 1). However, in directions where the roughness-element frontal area is reduced, ${Kan}_{z_0}$ can be up to 0.1 m less than ${Mac}_{z_0}$. Maxima between 120^o^ – 190^o^ and 270^o^ – 280^o^ are because of the taller houses. When wind flow is aligned with the smoother surface of the road to the west (210^o^ – 250^o^) values are lower. Morphometrically determined *z*_*0*_ are within the anemometric range, except when increased friction velocity from nearby roughness elements creates an anemometric *z*_*0*_ that is double the morphometric results (Fig. 6b, 120^o^ – 180^o^).

**Fig. 7:**
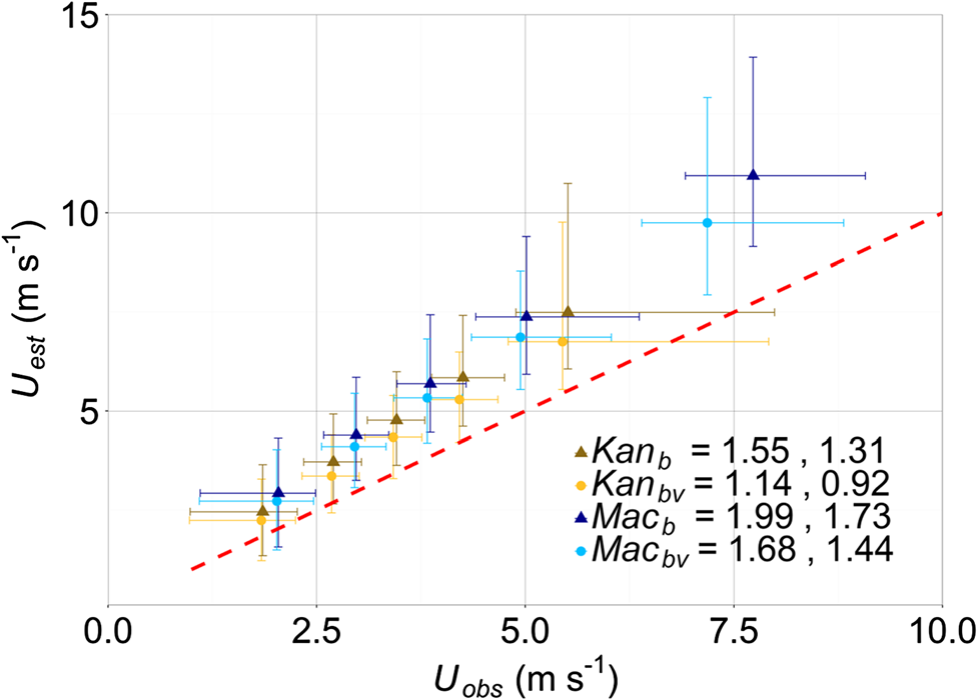
Observed (*U*_*obs*_) and estimated (*U*_*est*_, Eq. 10) wind speeds for each 30-min period (at the SWD site) using the Kanda et al. (2013, *Kan*) and Macdonald et al. (1998, *Mac*) morphometric methods, considering both buildings and vegetation (subscript *bv*) and only buildings (subscript *b*). Data are binned from lowest wind speed in groups of 1250 (30-min) data points. Median (points) and 5th and 95th percentiles shown. The root-mean-square error then mean absolute error (m s^−1^) (between *U*_*est*_ and *U*_*obs*_) are given in the legend

When the morphometric methods are applied without considering vegetation, the average reduction of *z*_*d*_ is 0.3 m and *z*_*0*_ is 0.1 m (Fig. 7b, *Kan*_*b*_ and *Mac*_*b*_). However, these values are directionally dependent. For example, not considering the taller and more abundant vegetation between 030^o^ – 090^o^ means *z*_*d*_ and *z*_*0*_ are reduced with an average of up to 20% and 40%, respectively, for both morphometric methods. Giometto et al. (2017) also demonstrate overlooking vegetation leads to a reduction of up to 50% in both *z*_*d*_ and *z*_*0*_ for a neighbourhood site with a larger plan area of vegetation and taller trees than the SWD site. Both results highlight the importance of considering vegetation during aerodynamic parameter determination.

#### Similarities in aerodynamic parameter analysis between the sites

The variability in the anemometric methods and the interdependence of *z*_*d*_ and *z*_*0*_ (i.e. the former is used when determining the latter, Eq. 3) means it is difficult to use the anemometric methods as a basis for the most appropriate magnitude of *z*_*d*_ and *z*_*0*_. However, there are apparent similarities from the aerodynamic parameter analysis performed at both sites. There is greater uncertainty in the application of the temperature variance method to determine *z*_*d*_, than the wind variance method. The RMSE between *ϕ*_*est*_ and *ϕ*_*obs*_ of the former are consistently twice the latter, which is attributed to the thermal inhomogeneity of both sites and dissimilarity of roughness length between momentum and heat. Greater uncertainty was also found in the temperature variance method in a central urban area (Kent et al. 2017b).

As with previous applications, *z*_*d*_ determined using the temperature and wind variance methods at both sites indicates *z*_*d*_ is larger than *H*_*av*_ (e.g. Grimmond et al. 1998, 2002, Feigenwinter et al. 1999, Kanda et al. 2002, Tsuang et al. 2003, Christen 2005, Chang and Huynh 2007, Tanaka et al. 2011, Kent et al. 2017b). Additionally, morphometric *z*_*d*_ results are consistently smaller than anemometric results. However, the direct incorporation of height variability in ${Kan}_{z_d}$ means it is more similar to the anemometric methods than ${Mac}_{z_d}$. ${Mac}_{z_d}$ is less than *H*_*av*_ and may be appropriate for homogeneous groups of roughness elements. However, recent literature demonstrates that the disproportionate amount of drag imposed by taller roughness elements in a heterogeneous mix means *z*_*d*_ may indeed become larger than *H*_*av*_ (Jiang et al. 2008, Xie et al. 2008, Hagishima et al. 2009, Zaki et al. 2011; Millward-Hopkins et al. 2011, Tanaka et al. 2011, Kanda et al. 2013). This is particularly evident during leaf-on conditions at the SFP site (Fig. 5a), where *z*_*d*_ appraoches 2*H*_*av*_.

The contrast in magnitude of aerodynamic parameters determined using the *Kan* and *Mac* morphometric methods (${Kan}_{z_d}$ is typically twice ${Mac}_{z_d}$) relates to the *Kan* method’s direct consideration of roughness-element height variability. However, as the *Kan* method, is developed from *Mac*, their directional variability is similar. At both sites, the morphometric methods show similar directional variability to the anemometric methods, indicating sound performance of Kent et al.’s (2017a) vegetation parameterisation.

For both morphometric methods, the range of *z*_*d*_ and *z*_*0*_ for any direction is consistently within ±1 m and 0.2 m of the median, respectively (Fig. 5 shading and Fig. 6 range of points for a direction). The range is attributed to the source area variability with meteorological conditions. For example, a wider range between 000^o^ – 120^o^ at the SFP site is caused by the proportion of the source area which falls upon the lake, grassed area and surrounding trees (Fig. 5).

### Implications for wind-speed estimation

With pre-determined *z*_*d*_ and *z*_*0*_, the logarithmic wind law can be used to model the neutral vertical profile of wind speed (Tennekes 1973):

10 $$ {\overline{U}}_z=\frac{u_{\ast }}{\kappa}\ln \left(\frac{z-{z}_d}{z_0}\right) $$

which theoretically only applies in the inertial sublayer (ISL), where vertical fluxes of momentum can be assumed constant with height (e.g. Tennekes 1973). Closer to a rough surface (i.e. within the RSL) the roughness-element wakes create a highly variable flow which may deviate considerably from Eq. 10 (e.g. Thom et al. 1975, Simpson et al. 1998, Kastner-Klein and Rotach 2004, Christen 2005, Harman and Finnigan 2007, Barlow and Coceal 2009, Giometto et al. 2016). With measurements at the SFP site (1.6*H*_*av*_) closer to the roughness elements, there is greater confidence to use Eq. 10 at the SWD site (where *z*_*m*_ = 2.8*H*_*av*_).

To assess the vegetation parameterisation within the *Kan* and *Mac* morphometric methods, the wind speeds measured at the SWD site during neutral conditions (|*z’/L*| < 0.05) are estimated using Eq. 10 with the observed *u*_∗_ for each 30-min period and roughness parameters determined with, and without vegetation (Fig. 6, subscript *bv* with and *b* without vegetation). The estimated wind speed (*U*_*est*_) is regressed against the mean observed wind speed (*U*_*obs*_) for the corresponding time period (Fig. 7). As the RMSE has been demonstrated to disproportionately amplify the error associated with outliers when assessing model performance (Willmott and Matsuura 2005), both the RMSE and mean absolute error (MAE) between *U*_*est*_ and *U*_*obs*_ are reported.

Wind speeds are overestimated in over 90% of cases, which is more apparent at higher *U*_*obs*_ (Fig. 7). Overestimation could be for several reasons, including uncertainty of the use of the logarithmic wind law closer to roughness elements or the appropriateness of *z*_*d*_ and *z*_*0*_ values obtained from the different methods (e.g. Millward-Hopkins et al. 2012). However, irrespective of the morphometric method *U*_*est*_ most resembles *U*_*obs*_ when aerodynamic parameters determined considering vegetation and buildings are used. For example, wind speeds estimated using *Kan*_*bv*_ and *Mac*_*bv*_ have MAE from *U*_*obs*_ of 0.92 and 1.44 m s^−1^, respectively, whereas ignoring vegetation (i.e. *Kan*_*b*_ and *Mac*_*b*_) the MAE is > 0.3 m s^−1^ larger for both methods (1.31 and 1.73 m s^−1^, respectively). The lower errors (both RMSE and MAE) associated with the *Kan* method indicate that regardless of whether vegetation is considered, incorporating height variability improves wind-speed estimates.

**Fig. 8:**
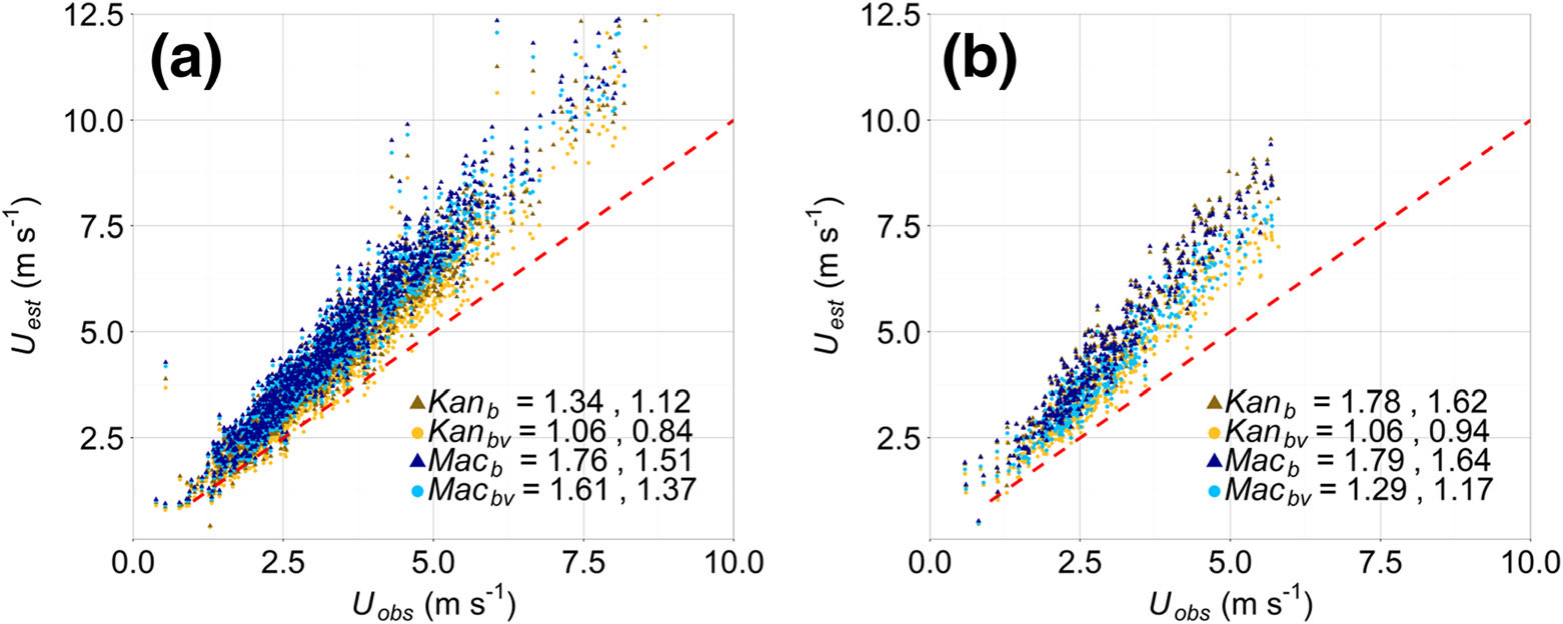
As for Fig. 7, but for wind directions between: **(a)** 210^o^ – 240^o^ and **(b)** 030^o^ – 090^o^. Each point represents a 30-min period of observations (data are not binned)

Similar comparisons between *U*_*est*_ and *U*_*obs*_ are performed for wind directions with the least (210^o^ – 240^o^) and greatest (030^o^ – 090^o^) vegetated roughness elements (Fig. 8a and b, respectively). As the least vegetated directions have similar aerodynamic parameters (Fig. 6) their associated *U*_*est*_ are similar irrespective of whether vegetation is considered or not. However, despite the small number of trees, accounting for them still reduces the error in wind speed estimation (i.e. the lower errors of *Kan*_*bv*_ and *Mac*_*bv*_) (Fig. 8a). The importance of considering height variability is apparent again, as the *Kan* method reduces the errors from *U*_*obs*_ by over 0.5 m s^−1^, in comparison to the *Mac* method.

As expected, in directions with maximum vegetation (tree) cover (030^o^ – 090^o^) the impact on estimated wind speeds is greatest. Inclusion of vegetation consistently results in an improvement of wind-speed estimation of over 0.5 m s^−1^ (Fig. 8b). The smallest differences between the errors associated with *Kan*_*bv*_ and *Mac*_*bv*_ occur in this direction (0.2 m s^−1^). Combined with the errors for *Kan*_*b*_ being larger than *Mac*_*b*v_, the incorporation of vegetation appears more important for accurately estimating the wind speeds than considering height variability (in this case).

## Conclusions

Two anemometric and two morphometric methods are used to determine the zero-plane displacement (*z*_*d*_) and aerodynamic roughness length (*z*_*0*_) for an urban park and a suburban neighbourhood. The anemometric methods use *in-situ* single-level high frequency observations and therefore inherently include the presence and state of vegetation. The morphometric methods have been developed for bluff bodies only, however a new parameterisation (Kent et al. 2017a) to consider both buildings and vegetation is explored.

At both sites, *z*_*d*_ determined using the anemometric methods is larger than the morphometric methods. There is greater uncertainty in an anemometric method based upon scaled temperature variance, as opposed to the vertical wind velocity variance, likely because of the thermal inhomogeneity of the sites. However, the Kanda et al. (2013) morphometric method, which directly considers roughness-element height variability, is consistently most similar to observations, indicating *z*_*d*_ is larger than average roughness-element height at the respective sites.

Inter-seasonal analysis is performed at the urban park, which is predominantly vegetation, with few buildings. Both anemometric methods indicate *z*_*d*_ during leaf-on vegetation state is up to 1–4 m larger than leaf-off. In addition, leaf-on *z*_*d*_ is obviously larger in directions with taller, or a greater proportion of, vegetated roughness elements. The morphometric methods with the vegetation parameterisation have a similar magnitude and directional variability of change, indicating leaf-on *z*_*d*_ is 1–3 m larger than leaf-off, which varies with upwind roughness elements. When the anemometric *z*_*d*_ is similar to, or larger than, the measurement height *z*_*0*_ cannot be determined from observations. However, the morphometric methods indicate leaf-on *z*_*0*_ may be less than half leaf-off *z*_*0*_ because the additional tree foliage in an already densely packed area creates an effectively smoother canopy.

The suburban neighbourhood has a larger proportion of buildings than trees. Morphometric analyses are undertaken during leaf-on conditions with and without vegetation. Where there is confidence in the anemometric methods, their *z*_*d*_ and *z*_*0*_ can be directly related to surface characteristics surrounding the site. The morphometric methods have similar directional change to the anemometric methods, but with less variability as the geometry of the site surroundings are similar. If vegetation is ignored in the morphometric calculations, *z*_*d*_ and *z*_*0*_ decrease by to 20% and 40%, respectively.

Wind speeds estimated at the suburban site using the logarithmic wind law and aerodynamic parameters from the morphometric methods are compared to observed wind speed at approximately three times the average roughness-element height. Wind-speed estimations most resemble observations when vegetation (in addition to buildings), as well as the height variability of roughness elements are considered. The consideration of vegetation is more important than the roughness-element height variability in directions where vegetation cover is maximal.

Kent et al.’s (2017a) extension of the morphometric methods captures the presence and state of vegetation for aerodynamic parameter determination and wind-speed estimation. As green spaces become increasingly part of the urban fabric, understanding the implications of vegetation upon aerodynamic characteristics becomes more important. Further observations with different types, amounts and arrangements of vegetation will allow more thorough assessment of this parameterisation.

The methodology to determine *z*_*d*_ and *z*_*0*_ from surface elevation databases (including vegetation) is freely available in the Urban Multi-scale Environmental Predictor (UMEP, http://www.urban-climate.net/umep/UMEP) for the open source software QGIS.
